# Telomere Length as a Quantitative Trait: Genome-Wide Survey and Genetic Mapping of Telomere Length-Control Genes in Yeast

**DOI:** 10.1371/journal.pgen.0020035

**Published:** 2006-03-17

**Authors:** Tonibelle Gatbonton, Maria Imbesi, Melisa Nelson, Joshua M Akey, Douglas M Ruderfer, Leonid Kruglyak, Julian A Simon, Antonio Bedalov

**Affiliations:** 1 Clinical Research Division, Fred Hutchinson Cancer Research Center, Seattle, Washington, United States of America; 2 Department of Genome Sciences, University of Washington, Seattle, Washington, United States of America; 3 Lewis-Sigler Institute for Integrative Genomics and Department of Ecology and Evolutionary Biology, Princeton University, Princeton, New Jersey, United States of America; 4 Human Biology Division, Fred Hutchinson Cancer Research Center, Seattle, Washington, United States of America; 5 Department of Medicine, University of Washington, Seattle, Washington, United States of America; Yale University, United States of America

## Abstract

Telomere length-variation in deletion strains of Saccharomyces cerevisiae was used to identify genes and pathways that regulate telomere length. We found 72 genes that when deleted confer short telomeres, and 80 genes that confer long telomeres relative to those of wild-type yeast. Among identified genes, 88 have not been previously implicated in telomere length control. Genes that regulate telomere length span a variety of functions that can be broadly separated into telomerase-dependent and telomerase-independent pathways. We also found 39 genes that have an important role in telomere maintenance or cell proliferation in the absence of telomerase, including genes that participate in deoxyribonucleotide biosynthesis, sister chromatid cohesion, and vacuolar protein sorting. Given the large number of loci identified, we investigated telomere lengths in 13 wild yeast strains and found substantial natural variation in telomere length among the isolates. Furthermore, we crossed a wild isolate to a laboratory strain and analyzed telomere length in 122 progeny. Genome-wide linkage analysis among these segregants revealed two loci that account for 30%–35% of telomere length-variation between the strains. These findings support a general model of telomere length-variation in outbred populations that results from polymorphisms at a large number of loci. Furthermore, our results laid the foundation for studying genetic determinants of telomere length-variation and their roles in human disease.

## Synopsis

Telomere maintenance is of great importance to ensure genome stability in organisms with linear genomes. In humans, telomeres shorten as a function of age and serve as a marker of cell replication history. Understanding the genetic differences in telomere length-maintenance may help provide the insights into the basis for different rates of aging among individuals and differences in individuals' propensity for aging-associated diseases such as cancer. Studies in yeast and other model organisms have defined several pathways that ensure stability of chromosome ends. In order to capture full complement of genes that participate in telomere maintenance in yeast *Saccharomyces cerevisiae,* the authors undertook a comprehensive screen for genes that affect telomere length. Among 152 identified genes, the authors found 39 genes whose function is critical for telomere maintenance in the absence of telomerase. The authors extended their studies from laboratory yeast strains to outbred populations of yeast and discovered significant phenotypic variation in telomere length among the isolates. Telomere length-analysis of a cross between a wild yeast isolate and a laboratory strain support a general model of telomere length-variation in outbred populations that results from polymorphisms at a large number of loci. This finding provides a basis for genetic studies of telomere maintenance in human populations.

## Introduction

Telomeres are complex structures at the ends of linear chromosomes composed of DNA, proteins, and ribonuclear protein complexes [1]. Telomeric DNA is composed of highly repetitive sequences (T_2_AG_3_)_n_ in humans and (C_1–3_A/TG_1–3_)_n_ in yeast [2]. The primary function of telomeres is to prevent genomic instability by ensuring complete DNA replication and protecting ends of chromosomes. While repetitive sequences in yeast telomeres span an average of 350 base pairs (bp), human telomeres exceed several kilobases (kb) [3]. In all organisms studied, telomere length can vary significantly from the equilibrium value and still support viability and replicative proficiency, indicating that there is considerable leeway in the absolute number of telomeric repeats required to maintain telomere functions.

Several pathways have been identified that regulate telomere length in yeast and humans. Telomerase is a highly specialized ribonuclear reverse transcriptase enzyme that catalyzes extension of 5′-ends of the lagging DNA strand using an RNA template [4]. Yeast telomerase is composed of the reverse transcriptase catalytic subunit (Est2p), an RNA template (TLC1) [5], and two additional protein subunits (Est1p and Est3p) [6]. Telomerase activity can overcome telomere shortening that results from the “end-replication problem” [7,8]. In humans, telomerase activity occurs only in germ cells and a subset of proliferating somatic cells [9]. As a result, in most human cells telomeres shorten as a function of cellular division and serve as a genetic and biochemical clock of cellular replication [10]. In yeast, the absence of telomerase leads to replicative senescence after 60–80 doublings [11]. Related telomere erosion-induced senescence phenotypes [10] have been observed in human cells in culture, which raised the possibility that the process of telomere erosion may contribute to cellular and organismal aging in humans [12]. Additional pathways involved in telomere length- regulation in yeast include telomere- or telomerase-interacting proteins (Rap1p, Rif1p, Rif2p, and Pif1p), the Ku70/Ku80 end-capping complex [13], the nonsense-mediated RNA decay (NMD) pathway (Nmd2p, Upf3p, and Nam7p) [14], and the RMX (Rad50p, Mre11p, Xrs2p) DNA-strand break repair complex [15]. The genetic and functional relationships among these diverse pathways are under active investigation [16]. In this study, we used a collection of yeast-deletion strains in order to capture full complement of non-essential genes that control telomere length in yeast and examined their relationship with major telomere-maintenance pathways. Furthermore, we evaluated the genetic basis for telomere length-variation in outbred yeast populations.

## Results

### Screening Deletion Strains for Genes That Regulate Telomere Length

**Figure 1:**
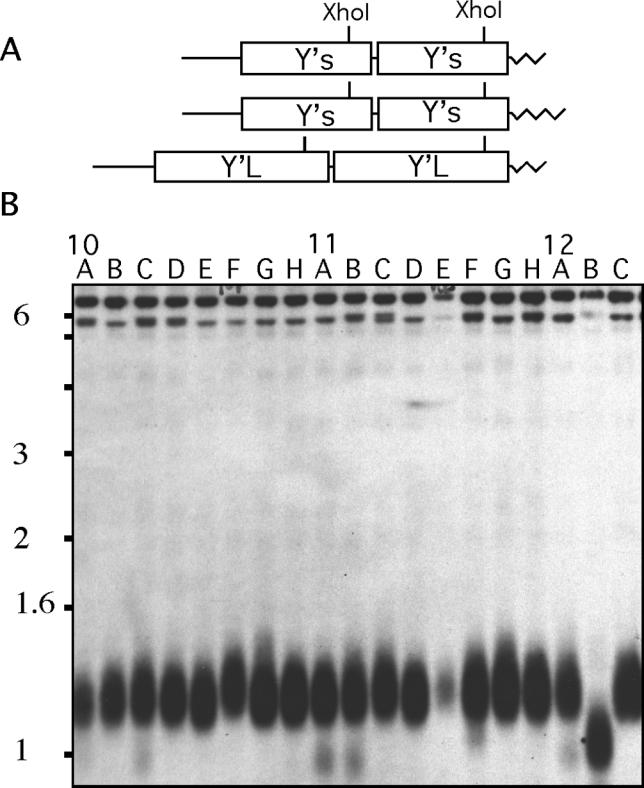
Telomere and Subtelomeric Repeats in S. cerevisiae (A) The average length of imperfect (C_1–3_A/TG_1–3_)_n_ telomeric repeats is 350 bp. Y′-s and Y′-L are subtelomeric repetitive elements 5.2 and 6.7 in length. (B) A representative Southern blot from the telomere length survey. Terminal restriction fragments are visualized using Y′-probe. Short terminal restriction fragment in lane 12B corresponds to *rad50Δ*.

We carried out a screen of 4,820 *MATa* haploid deletion strains representing the majority of non-essential yeast genes to identify loci that contribute to telomere maintenance. DNA was isolated from each strain, digested with XhoI restriction enzyme, and after electrophoresis, analyzed by Southern blotting using a Y′-subtelomeric probe. Most yeast chromosome ends contain one or several Y′-repetitive elements [17]. The most distal XhoI site on the chromosome is located close to the 3′-end of the Y′-element so that the terminal restriction fragment contains about 900 bp of the subtelomeric Y′-repeat and approximately 350 bp of telomeric TG repeats (Figure 1A). In addition, the use of Y′-probe resolves two major large restriction fragments derived from the tandem repeats of longer Y′- (6.7 kb) and shorter Y′- (5.2 kb) elements. There are several other minor bands that vary in length due to the presence of other repetitive sequences such as X′-elements. A representative Southern blot from the initial screen is shown in Figure 1B and the blots for all the deletion strains are provided as supplemental data at http://www.fhcrc.org/labs/bedalov/index.html. The magnitude of the telomere length-alteration was scored on a 1–3 scale: (1) in short telomere strains corresponding to the reduction of the telomere length by 200, (2) 200–50, or (3) less than 50 bp; and in long telomere strains, (1) an increase by more than 300, (2) 50–300 bp, or (3) less than 50 bp.

There were 247 strains in the haploid *MATa* set identified as having altered telomere length in the initial screen and further analyzed by re-isolating their DNA and confirming the telomere length-alteration. If confirmed, telomere length in the corresponding homozygous diploid mutant was analyzed. Only mutants that had consistent telomere length- alterations in haploid and homozygous diploids were used for further analysis. As the next step in identifying telomere length-maintenance *(TLM)* genes, we carried out allelism analysis of the *KanMx* locus and the altered telomere length for all the short telomere mutants and for selected long telomere mutants (mutants that scored 1 and 2). Allelism testing was performed through random spore and tetrad analysis of the progeny derived from the diploids generated by mating mutants with the wild-type strain and confirming the co-segregation of altered telomere length and the *KanMx* marker gene. Random spores were obtained through genetic selection for haploid *MATa KanMx* progeny using a strategy developed by Tong et al. [18] and analyzed for telomere length individually or as pools of spores. If the results of random spore analysis suggested lack of co-segregation of the *KanMx* and telomere length-alteration, tetrad dissections were carried out and examined for 2:2 co-segregation of the telomere length-change and *KanMx* marker. This battery of tests reduced the number of genes that regulate telomere length to 152 genes as listed in Table 1.

**Table 1:**
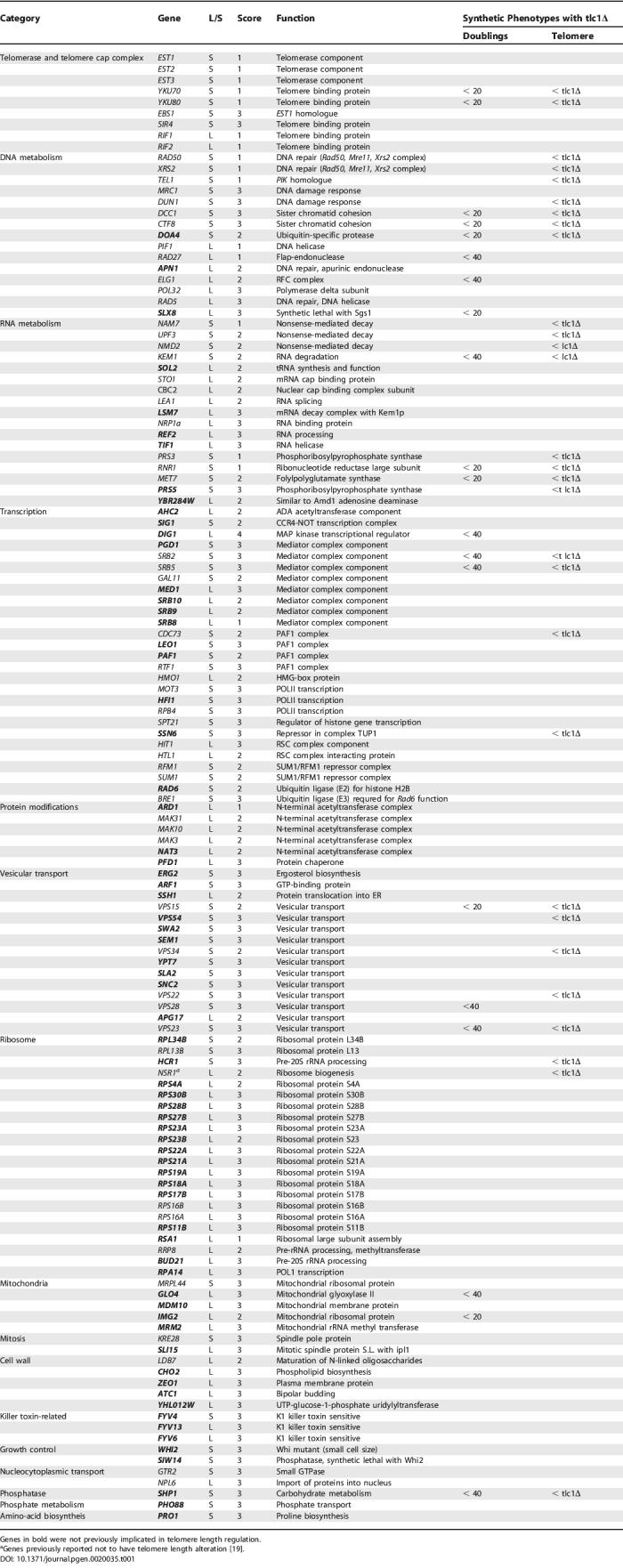
Genes Whose Deletion Affects Telomere Length and Their Interaction with Telomerase Pathway

**Figure 2:**
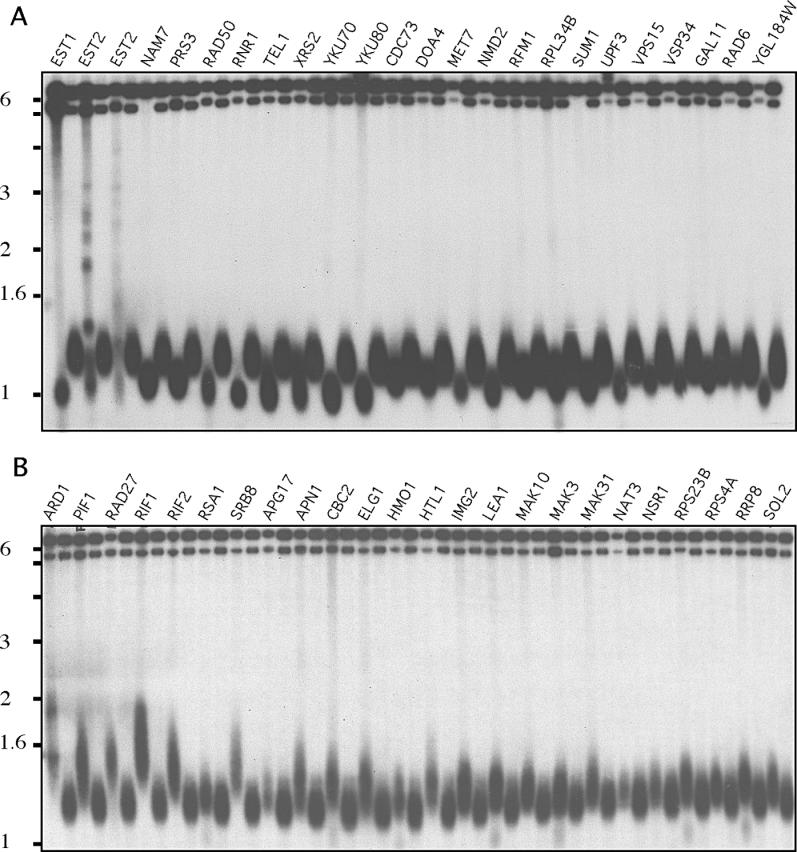
Telomere Blots of Mutants with Short and Long Telomeres Short (A) and long (B) telomeres. Every other lane (unlabeled) presents DNA isolated from wild-type cells.

Among 4,820 deletion mutants we found 72 *tlm* mutants with consistently short telomeres and 80 mutants with long telomeres (Figure 2 and Supporting Information). Of these mutants, 64 (42 with short and 22 with long telomeres) corresponded to genes previously identified as telomere maintenance genes including all three ever shorter telomere *(EST)* genes, providing validation for our screen. In addition to having the shortest telomeres in the deletion set, mutants in *EST* genes also exhibited a large variation in the size of the distal restriction fragment, a consequence of recombination and amplification of subtelomeric Y′-elements. No other *tlm* gene deletion exhibited this phenotype suggesting that there are no novel *EST* genes in the deletion set. Further validation came from a recent study, Askree et al., who reported a telomere length survey of the deletion set similar to ours [20]. While there is a significant overlap between the genes found to regulate telomere length in that study with the genes reported here, 94 are unique to our study.

### Relationship between *TLM* Genes and Major Telomere Length-Maintenance Pathways

*TLM* genes belonged to several functional classes: genes required for *POL2* transcription (27 genes) and ribosome biogenesis (22 genes); vacuolar protein sorting (VPS) (15 genes) represented the largest groups of genes. In order to begin to assign specific telomere maintenance roles to the newly identified genes, we examined their relationship with genes that perform known functions at telomeres: telomerase *(TLC1)* and telomere-capping functions *(YKU70)* [21].

Two assays were used to determine the relationship between the novel genes and telomerase. First, we examined whether the mutations affect the ability of yeast to replicate in the absence of telomerase (replicative senescence). Accelerated replicative senescence was interpreted as evidence for non-epistasis with the telomerase pathway. Second, we measured telomere length in double mutants that lack the *TLM* gene and *TLC1,* the RNA component of telomerase. The *TLM* genes that participate exclusively in the telomerase pathway are not expected to affect replicative senescence or increase the rate of telomere loss in the absence of telomerase. Conversely, if misregulation of telomerase activity is responsible for the phenotype in mutants that have increased telomere length, the increased telomere length will be entirely dependent on telomerase.

**Figure 3:**
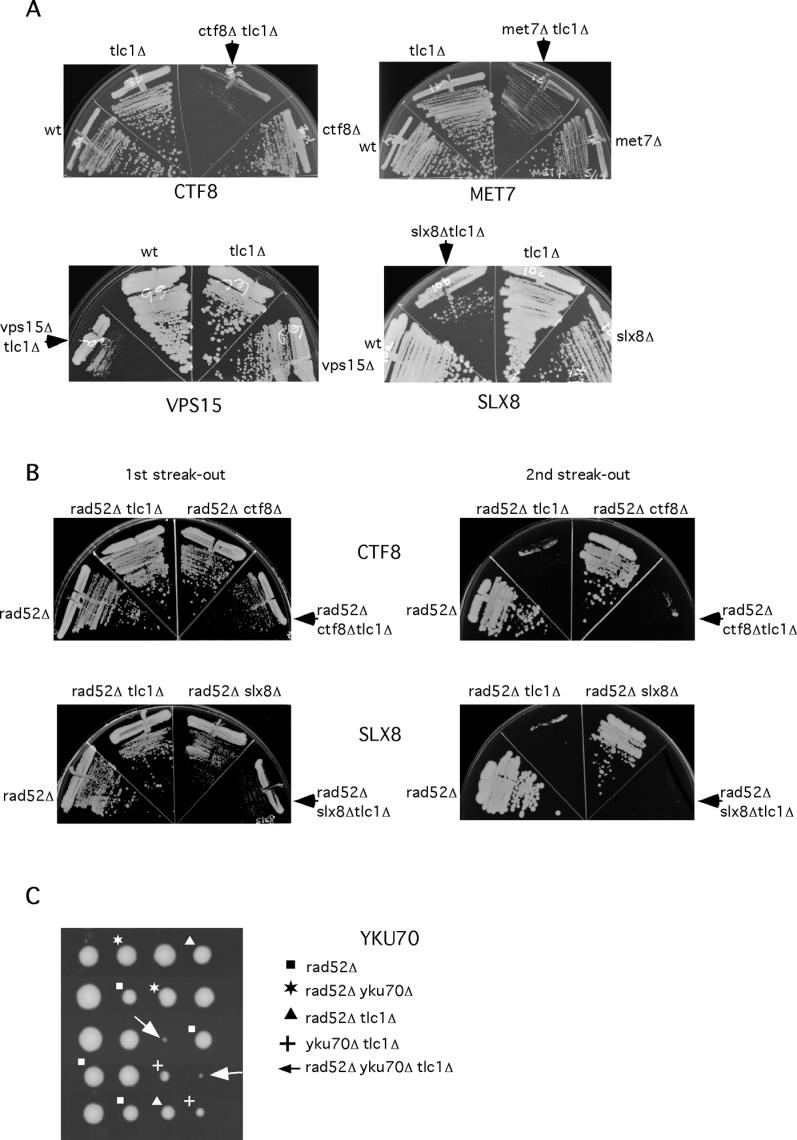
*TLM* Genes That Have Synthetic Phenotypes with the Lack of Telomerase (A) Senescence assay. Following sporulation, a *TLC1/tlc1 TML/tlm* heterozygous diploid strain and spore germination, haploid single *tlc1, tlm,* and double *tlc1 tlm* mutants and wild-type progeny derived from the same tetrad were streaked-out on rich medium. Strains with double mutations grew poorly relative to *tlc1* and *tlm* single mutation. Genes required for chromatid cohesion (e.g., *CFF8*), dNTP biosynthesis (e.g., *MET7*), VPS (e.g., *VPS15*), and DNA replication (e.g., *SLX8*) are required for growth in the absence of telomerase. (B) *TLM* genes affect senescence rate independently of *RAD52*-mediated homologous recombination pathway. Spores derived from *TLC1/tlc1 TML/tlm RAD52/rad52* heterozygous diploid strains were allowed to germinate, and haploid single *rad52,* double *rad52 tlc1, rad52 tlm,* and triple *rad52 tlc1 tlm* mutants were streaked-out successively on rich medium. The strains with triple mutations *(rad52 tlc1 tlm)* grew poorly relative to strains containing *rad52 tlc1* double mutations. (C) Synthetic lethality induced by the lack of *RAD52, TLC1,* and *YKU70.* Tetrads were derived from *TLC1/tlc1 YKU70/yku70 RAD52/rad52* heterozygous diploid strain. Unlike the double *tlc1 yku70, tlc1 rad52,* or *yku80 tlc1* mutants that are capable of colony formation (note that *yku70 tlc1* double mutants form small colonies), the triple *rad52 tlc1 yku70* mutant spores die as microcolonies.

Growth of telomerase-deficient yeast is characterized by gradual loss of telomeric DNA that is accompanied by loss of viability or senescence. After 60–80 doublings, telomerase-deficient *tlc1* cells stop dividing except for a few survivors that have gained the capacity to maintain their telomeres through homologous recombination [6,22]. We carried out senescence assays by performing several successive streak-outs of single *tlc1* and double *tlc1 tlm* mutants. As previously reported, senescence phenotype of *tlc1 est1* double mutant was similar to that of *tlc1* or *est1* single mutants. However, double *tlc1 yku70* mutants known to be defective in the telomerase-capping functions senesce after approximately 20 divisions in the absence of telomerase (Table 1 and unpublished data). We identified 22 *tlm* (15 short-telomere and seven long-telomere) genes whose loss accelerated senescence of telomerase-deficient yeast. *TLM* genes in this category included those that participate in sister chromatid cohesion (e.g., *DCC1, CTF8*), deoxyribonucleotide (dNTP) biosynthesis *(MET7, RNR1),* VPS *(VPS15, VPS28, VPS23),* transcription *(SRB2, SRB5),* and DNA replication and repair *(SLX8, ELG1, RAD27, DOA4)* (Figure 3A and Table 1). Furthermore, mutants in two genes with unknown functions, *YDL119* and *YPL017,* also exhibited a more rapid loss of viability in the absence of telomerase. These results suggest that a subset of *tlm* genes carry out functions at telomeres that are not exclusively dependent on telomerase.

Because homologous recombination is important for telomere maintenance and cell proliferation in the absence of telomerase [6,22], we examined the possibility that *TLM* genes affect senescence through interfering with *rad52*-dependent homologous recombination pathway. We therefore compared the senescence rates of *tlc1 rad52* double and *tlc1 rad52 tlm* triple and double mutants for several *TLM* genes including *YKU70, CTF8, SLX8, DCC1, MET7,* and *YPL017c.* As reported previously [6], we found that *tlc1 rad52* double mutants lose viability after approximately 40 doublings (i.e., most of the *tlc1 rad52* double mutants are incapable of forming colonies upon second streak-out) (Figure 3B). The lack of *TLM* genes further accelerate loss of viability, as *tlc1 rad52 tlm* triple mutants die as microcolonies following germination (e.g., *yku70*) (Figure 3C) or die at the first streak-out (e.g., *ctf8, slx8, dcc1, ypl017c,* and *met7*) (Figures 3B and S1). The observation that the senescence rate effect of *TLM* genes is preserved in the absence of *RAD52* suggests that homologous recombination is not the primary pathway through which described *TLM* genes affect senescence.

**Figure 4:**
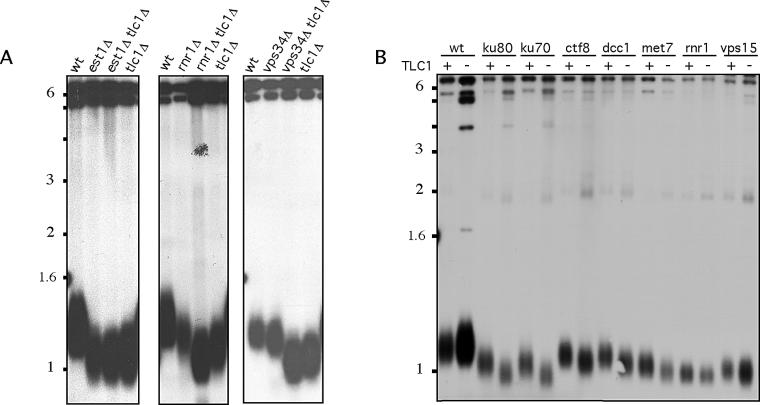
The Lack of *TLM* Genes Accelerates Telomere Loss in the Absence of Telomerase (A) Mutants in genes required for dNTP synthesis *(RNR1)* and VPS *(VSP34)* exhibit accelerated telomere loss in the absence of telomerase. This shows telomere length of single *tlc1, tlm,* and double *tlc1 tlm* mutants 20 doublings after germination of *TLC1/tlc1 TML/tlm* heterozygous diploids. Telomere length in *est1 tlc1* double mutants is the same as the telomere length in *tlc1* or *est1* single mutants. Telomere length of double *rnr1 tlc1* and *vps34 tlc1* mutants is shorter than the telomere length of single *rnr1, vps34,* or *tlc1* singe mutants. (B) The loss of telomeric DNA in the absence of telomerase is accelerated in dNTP *(rnr1, met7),* sister chromatid cohesion *(ctf8, dcc1),* and VPS *(vps15)* mutants. Single *tlc1* or double *tlc1 tlm* mutants containing galactose-inducible TLC1 plasmid (GAL-TLC1) were grown in galactose medium (TLC1 +) or in glucose (TLC −) medium for five cell divisions. Single *tlc1* mutants do not have significant telomere loss while the telomere loss is easily appreciated in the double *tlc1 tlm* mutants.

To evaluate the relationship between telomerase and other telomere maintenance genes with a more sensitive assay, we compared telomere length in the double *tlc1 tlm* mutant with the single *tlc1* mutant. In agreement with the synthetic interaction in the senescence assay, deletion of sister chromatid cohesion genes (e.g., *DCC1, CTF8*), dNTP biosynthesis *(RNR1, MET7, PRS3),* VPS, and two members of mediator complex *(SRB2* and *SRB5)* showed a synthetic telomere phenotype (reduced telomere length) with the lack of *TLC1* (Figure 4A and Table 1). This result confirms that more rapid loss of viability in this subset of double mutants is directly related to accelerated loss of telomeric DNA. However, synthetic interaction was also seen with members of other groups, which did not show an accelerated senescence phenotype, including all of the nonsense-mediated decay (NMD) pathway genes (e.g., *NAM7, UPF3, NMD2*), telomeric DNA-end-processing *(XRS2, RAD50),* and two ribosomal genes *(RPL13B* and *RPL34B).* In order to directly examine the effect of *tlm* genes on the rate of telomere loss induced by the absence of telomerase, we employed a galactose-inducible telomerase system. Single *tlc1* and double *tlm tlc1* strains containing a galactose-inducible *TLC1* on a plasmid (GAL-TLC1) were grown in galactose medium (telomerase ON) and then switched to glucose medium (telomerase OFF). After five divisions without telomerase in glucose medium, telomere length of *tlc1* strain was indistinguishable from the telomere length of a strain grown in galactose, whereas the telomere length in the double *tlc1 tlm* mutants was reduced (Figure 4B). These results demonstrate that the rate of telomere loss in the absence of telomerase is accelerated in a subset of *tlm* mutants, which further confirms the role of the corresponding *TLM* genes in telomerase-independent telomere maintenance mechanisms.

**Figure 5:**
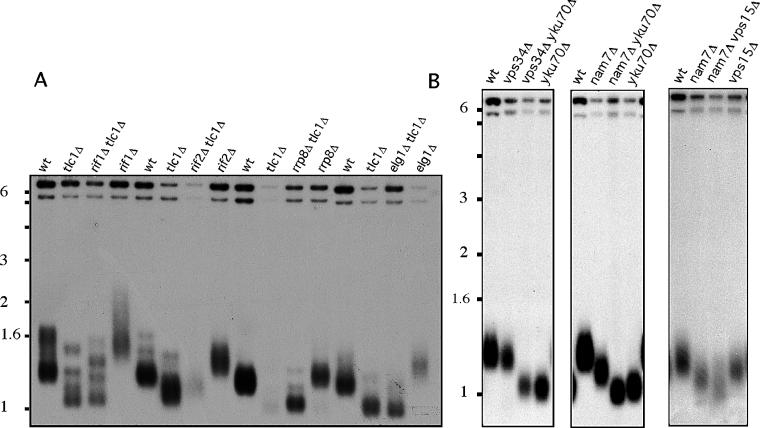
Epistatic Analysis of Telomere Length among *TLM* Genes (A) Telomerase is required for increased telomere length in long telomere mutants. This shows telomere length of single *tlc1, tlm,* and double *tlc1 tlm* mutants 20 doublings after germination of *TLC1/tlc1 TML/tlm* heterozygous diploids. Telomere length of double *tlc1 tlm* mutants is similar to telomere length of *tlc1* single mutants. (B) VPS genes and NMD genes participate in separate telomere-maintenance pathways. VSP genes (e.g., *VPS34, VPS15*) are epistatic with KU telomere-capping pathway (e.g., *YKU70*), and NMD genes (e.g., *NAM7*) have synthetic telomere phenotype with the lack of *YKU70.*

Similar analyses were carried out in a subset of mutants that confer long telomeres to establish whether their increased telomere length was dependent on telomerase activity (Figure 5A). Telomere length of the long telomere *tlm tlc1* double mutants was indistinguishable from the telomere length of the *tlc1* single mutant, indicating that their long telomere phenotype was entirely dependent on the telomerase pathway. This result is consistent with the model where the absence of *TLM* genes perturbs normal mechanisms that limit telomerase access to telomeres. Interestingly, several of the long telomere mutants exhibited accelerated loss of viability in the absence of telomerase (e.g., *rad27, slx8, elg1*) (Figure 5A and Table 1), suggesting that the same defect that increased telomerase-mediated telomere elongation in the presence of telomerase makes chromosome ends more vulnerable in the absence of telomerase.

The synthetic phenotype of NMD genes and VPS genes with the lack of telomerase raised the possibility that these mutants affect telomere capping. The Ku DNA-end-binding proteins carry on an important telomere-capping role. We therefore evaluated the relationship of KU pathways and NMD and VPS genes by comparing telomere length in single *yku70* and double *yku70 nmd* or *yku70 vps* strains. The loss of *YKU70* exhibited a synthetic phenotype with the loss of NMD genes and was epistatic with the loss of VPS genes (Figure 5B and unpublished data). This result indicates that VPS genes function in the KU-capping pathway distinct from the NMD pathway. In further support of this idea we observed synthetic telomere phenotypes between VPS and NMD genes (Figure 5B).

### Telomere Length-Variation in Natural Isolates

**Figure 6:**
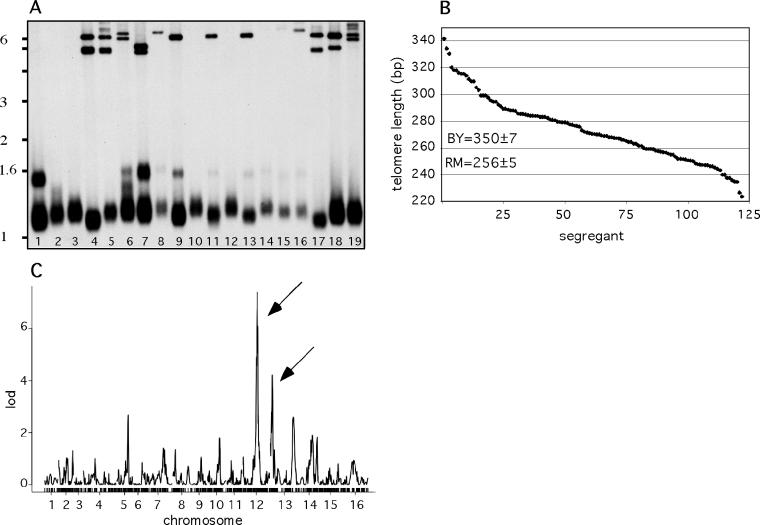
Telomere Length-Analysis in Outbred Yeast Strains (A) Variation of telomere length among natural isolates of *S. cerevisiae.* Southern blot with terminal restriction fragments from diploid strains (lanes 1–12, 14) isolated from vineyards, their haploid derivatives (lanes 13, 15–17), and two laboratory strains BY (lane18) and A363A (lane 19). Haploid strains RM5–1 (lane 13) are derived from diploid strain Bb24(4) (lane 9), haploid strain Y55a (lane 15) from diploid strain Y55 (lane 14), haploid strain RM3-1a (lane 16) from diploid strain Bb3(1) (lane 8), and haploid strain RM4–1 (lane 17) from diploid strain Bb24(3) (lane 4). Other diploid strains include Bb26(5) (lane 1), Bb31(2) (lane 2), Bb32 or RM11 (lane 3), PC25A (lane 5), Ba25 (lane 6), Bb2(1) (lane 7), Bb25(6) (lane 10), Bb 25(8) (lane 11), and Bb 26(2) (lane 12). (B) Average telomere length-variation in 122 haploid progeny of a cross between vineyard (RM11-1a, a haploid progeny of diploid RM11) and laboratory (BY) strain. Average telomere length of the progeny is continuously distributed between 222 and 342 bp, consistent with the genetic trait controlled by several loci. The average telomere length in the parents, obtained through three independent measurements, was 350 ± 7 and 256 ± 5 for BY and RM11, respectively. (C) Genome scan for loci that control telomere length in BY and RM11. The arrows indicate significant loci at Chromosome 12 and Chromosome 13.

Our results using the laboratory yeast strain indicate that myriad genes control telomere length. A trait that is controlled by a large number of genes has the potential to exhibit phenotypic variation in genetically diverse populations, and indeed, when we analyzed variation in telomere length in 13 Saccharomyces cerevisiae strains isolated from the wild [23], we found that the size of the terminal restriction fragment varied significantly among different strains (Figure 6A). The difference in size was due exclusively to alterations in the size of telomeric repeats and was not due to the variation in the size of the Y′-DNA as determined by the analysis of the terminal fragment by PCR and DNA sequencing (unpublished data). The size of the telomeric DNA between the isolates varied between 150 and 400 bp. We hypothesized that different telomere lengths between the strains were due to polymorphisms in many loci. To further evaluate this possibility, we analyzed telomere length in the progeny derived from crossing the laboratory strain (BY) and one of the wild isolates (RM11). The telomeres in the haploid RM11 strain are approximately 100 bp shorter than the telomeres in the haploid BY strain. In order to study the underlying genetic basis of this difference in telomere length, we analyzed telomere lengths of 122 haploid progeny from a cross between BY and RM11. The distribution has several interesting features (Figure 6B). First, the segregants show a continuous broad range of telomere lengths, consistent with a trait that is controlled by many loci. Second, the average length of telomeres among the segregants of 272 bp is shorter than the average length of the parental strains (302 bp), which is suggestive of non-additive interactions among the loci involved. Finally, it is interesting to note that none of the segregants has telomere length longer than BY; while a fraction (27%) have telomere length shorter than RM11. The phenomenon of a trait having progeny values more extreme than either parent, called transgressive segregation, is observed for many traits and indicates the presence of multiple loci that have compensating effects in the parents. Transgressive segregation can occur as a result of non-additive interactions among the loci and is consistent with the above observation. Furthermore, while all the segregants in this study shared the same replication history following germination (having undergone approximately 80 doublings), it is possible that final telomere length-equilibrium had not been achieved, which may skew the distribution of the telomere lengths.

To identify quantitative trait loci- (QTL) mediating variation in telomere length in the segregants, we performed a genome-wide linkage analysis. In this analysis, genetic markers were used to classify each locus in the genome as inherited from the BY or RM11 parent in each segregant. Then, for each locus, the distributions of telomere lengths were compared between segregants inheriting the *BY* allele and those inheriting the *RM11* allele. A significant difference between these distributions indicates that the tested locus lies near a sequence polymorphism between the two strains that affect telomere length. Specifically, linkage analysis was performed with 3,312 genetic markers that were previously genotyped in all of the segregants using oligonucleotide microarrays [24,25]. Telomere length showed evidence of linkage to several loci (Figure 6C), with significant linkage peaks occurring on Chromosome 12 and Chromosome 13 (Table 2). The LOD scores of 7.4 and 4.3 at Chromosome 12 and Chromosome 13 loci were significant (genome-wide corrected *p* < 0.01), as we did not observe any scores higher than four in the analysis of 100 permuted datasets. Several genes known to regulate telomere length reside in the mapped regions including *EST1, VPS34, RPS28B, ARV1,* and *VPS9* [20]. Polymorphisms that result in amino-acid substitutions in these genes (Table S1) are likely candidates that control telomere length in the segregants. Alternatively, telomere length might be determined by the polymorphisms in essential genes that reside in these regions (i.e., genes that were not evaluated directly in this study) or by polymorphisms in the regulatory elements. Interestingly, the telomere length-effect of the Chromosome 12 locus was in the opposite direction from the difference between the parents. In other words, the Chromosome 12 *RM11* allele conferred longer telomeres and the *BY* allele conferred shorter telomeres, even though the RM11 parent has shorter telomeres than the BY parent, which is consistent with transgressive segregation (Table 2). The Chromosome 12 and Chromosome 13 loci explained only 25% and 13% of the telomere length-variation among the segregants, respectively, suggesting the presence of several other unidentified loci that contribute to telomere length-variation. The average telomere length of the segregants closer to the shorter telomere length of the RM11 parent is surprising given the finding that the *RM11* alleles of the major Chromosome 12 locus confer longer telomeres. Other *RM11* alleles from the loci that were not mapped and that confer short telomeres may account for this finding. Other loci with smaller effects on telomere length may not have been mapped due to limited sample size. Furthermore, genes that participate in telomere length-regulation have complex, epistatic relationships, which is consistent with the evidence for non-additive effects from the distribution of telomere lengths and may decrease the statistical power of single-locus linkage analysis. Taken together, our results support the idea that telomere length in genetically diverse yeast strains behaves as a quantitative trait controlled by many genes.

**Table 2:**
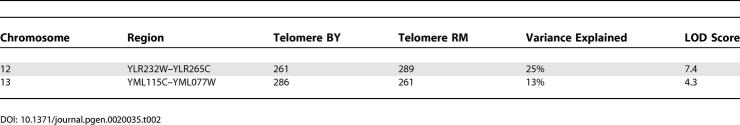
Loci That Control Telomere Length in RM and BY Strains

## Discussion

### Overview of the Telomere Length Screen

Askree et al. recently carried out a genome-wide survey of the haploid deletion strains of S. cerevisiae for alterations in telomere length and reported 119 mutants with short telomeres and 53 mutants with long telomeres [20]. In total, 58 genes overlap between Askree et al. and our data, leaving 114 genes unique to Askree et al. and 94 genes unique to our study. However, the results of the two screens are more congruent than these numbers imply, since both sets of telomere-associated genes include different members within the same genetic pathways. A striking example is the overlap in ribosomal protein gene deletions: 24 *RPS* and *RPL* genes were identified in the two studies (eight genes in Askree et al. and 16 genes in our study) with only two genes overlapping. Similar results were found with VPS genes and the members of mediator complex. Differences in technical details of the primary screens and different methods for verification of the initial hits likely account for the surprisingly low gene overlap and for considerable differences in the ratio of long to short telomere mutants in two studies. While XhoI restriction digest were used in both studies, Southern blots were carried out using a telomeric probe by Askree et al. and subtelomeric Y′-probe in our study. Unlike the Y′-probe, the use of telomeric probe is expected to increase the signal intensity of the long telomere mutants, which may be interpreted as DNA overloading rather than perturbation in telomere length and could be responsible for differences in the scoring of the mutants. On the other hand, the substantially lower number of the short telomere mutants in our study (72 versus 119) is likely due to more stringent criteria for verification of the initial hits, which in our study included evaluation of homozygous diploids as well as allelism studies for all short and some long telomere mutants (allelism studies were carried out for very small number of genes by Askree et al.). While assays in our study eliminated several of the genes reported to have strong telomere effect, in the study by Askree et al. (e.g., *CTK1, URE2*) the large majority of differences were in genes that were reported to have slight effects on telomere length. It should be noted that some telomere phenotypes require many cell divisions to fully manifest: since analyses of segregants were carried out after only 20–30 doublings in the mutants, some mutations that confer a delayed effect would not be detected creating a false-negative result.

The annotation of gene function for a large number of yeast genes illuminated several pathways involved in telomere maintenance. For example, within the polymerase II transcription group, which is the largest group of genes in our study, four separate pathways can be recognized: mediator complex (eight of the 11 non-essential members of the mediator complex show telomere length phenotypes), *PAF1* complex (four of five members) [26], *SUM1-RFM1-HST1* repressor complex (two of three genes) [27], and genes required for histone ubiquitylation *(RAD6, BRE1)* [28]. The phenotypes observed with mutants in this group tend to be mild (class 2 and 3) and may reflect an upstream effect on the master regulators of telomere length such as telomerase or end-capping.

Other functional groups represented with multiple mutants include VPS, ribosome biogenesis, and dNTP biosynthesis. Telomere length can be perturbed through alteration of any of several functions: telomeric DNA replication via telomerase and DNA polymerases, telomerase recruitment, DNA-end processing, and telomere-capping functions [3]. Many of the newly discovered telomere maintenance genes, including genes for dNTP biosynthesis, VPS, NMD, as well as several member of the mediator complex, show synthetic phenotypes with the lack of telomerase (i.e., accelerated senescence or increased telomere reduction), suggesting that they function in pathways outside telomere replication by telomerase. Our study and a recent work by Rog et al. [29] places VPS genes in the Ku70/Ku80 telomere-capping pathway and NMD genes in a separate telomere-capping pathway. Mutants in the NMD pathway were recently demonstrated to increase the levels of telomere-capping protein Stn1p [30], further supporting this model.

Accelerated senescence of mutants in the dNTP biosynthesis pathways demonstrates the importance of adequate levels of dNTPs for maintaining telomeric ends in the absence of telomerase, which is presumably most acute for lagging- strand synthesis. This finding is consistent with the recent results of Toussaint et al. [31], that short telomeres in thymidylate synthetase *(CDC21)* and thymidylate kinase *(CDC8)* mutants are not caused by the impairment in telomerase-mediated DNA elongation in the condition of lowered dNTP supply. Finally, the synthetic phenotypes of mutations in dNTP biosynthesis genes with the lack of telomerase suggest a therapeutic strategy that could increase the efficacy of telomerase inhibitors in anticancer chemotherapy. The concurrent use of drugs that alter dNTP pools (i.e., antimetabolites), together with telomerase inhibitors, may act synergistically and lead to more rapid telomere erosion and destruction of cancer cells.

### Telomere Length-Variation in Natural Isolates of S. cerevisiae and in Human Populations

Considerable length-variation found among the natural isolates of S. cerevisiae suggests the lack of evolutionary selection for any one particular telomere length and likely reflects subtle differences in telomere length-maintenance. Differences in telomere length have been previously observed among different laboratory yeast strains and attributed to genetic differences between the strains [32]. The total number of genes involved in telomere length-regulation encompasses approximately 3% of the yeast genome. Based on these findings, outbred yeast strains are expected to have a high number of polymorphisms giving rise to tremendous phenotypic variation. Human populations also exhibit considerable variation in telomere length. Telomere length was found to vary between five and 15 kb at birth. Twin studies indicated that 80% of telomere length-variation is genetically determined [33]. Telomeres progressively shorten as a function of age with the average loss of approximately 30 bp per year. It is possible that genetic differences in telomere length-maintenance, which are of no consequence early in life, contribute to different rates of aging among individuals and differences in individuals' propensity for aging-associated diseases such as cancer [34].

One of the crucial functions of telomeres is to protect chromosomes from end-to-end fusions and thus distinguish ends of chromosomes from DNA double strand breaks that need to be repaired. The role of telomere attrition in human carcinogenesis has been directly demonstrated by capturing telomere loss-induced chromosomal bridges in precancerous lesions in human colon [35] and breast [36]. Inter-individual differences in telomere metabolism might be reflected as different telomere length at birth or as different rates of telomere attrition during aging. Shorter telomeres in a population over the age of 60 have been correlated with increased overall mortality [37]. These findings highlight the relevance of genetic factors affecting telomere length-maintenance to aging and to human diseases. A detailed understanding of yeast telomere biology as well as a comprehensive list of genomic loci that affect telomere length makes S. cerevisiae a valuable system for studies of telomere length-variation. Our studies in yeast show that telomere length in outbred populations behaves as a quantitative trait. Linkage studies for loci that control telomere length in mice [38] led to identification of a helicase gene with an essential role in telomere maintenance [39]. Similar studies in human populations [40] hold promise to identify loci that determine telomere length and perhaps, predisposition to age-related diseases.

## Materials and Methods

### Yeast strains and media.

We used S. cerevisiae haploid and homozygous collections [41] of 4,820 strains in which each nonessential open reading frame has been replaced with the *KanMx* module that confers resistance to G418. The deletion strains were derived from BY4741 *MATa his3, leu2, met15,* and *ura3.*

The vineyard strains, collected and described by Robert Mortimer [23], were a gift from Lee Hartwell (Fred Hutchinson Cancer Research Center, Seattle, Washington, United States). The 122 segregants and their parent strains (S288C derivative BY4716 *MATa lys2*) and natural isolate derived from Bb32(2) (RM11-1a *MATa leu2, ura3, HO::KanMx*) were described [24,42].

### Analysis of telomere length.

Saturated culture (1.5 ml) of each deletion mutant strain was used for DNA extraction using alkaline lysis-based method in a 96-well format. DNA (5–10 μg) was digested with XhoI overnight and separated by electrophoresis (12–16 h at 1.5 V/cm using 0.95% agarose gel). After transfer of DNA to Hybond-N membranes (Amersham Biosciences, Little Chalfont, United Kingdom), terminal restriction fragments were visualized by hybridization with digoxigenin or ^32^P-labeled Y′-probes using chemilluminescence or autoradiography.

In order to analyze the length and sequence of subtelomeric Y′-DNA in vineyard and laboratory strains, terminal restriction fragment was PCR-amplified using a method devised by Myung et al. [43]. Briefly, nested Y′-sequence-specific primers were used together with telomeric repeat-specific primers in two rounds of PCR. The size of PCR products was analyzed using electrophoresis and the Y′-elements were sequenced from the second PCR product.

Linkage analysis of telomere length in the BY and RM11 cross was carried out using publicly available R/qtl software [44]. Genetic map of the segregants containing 3,312 markers covering 99% of the genome was previously generated using oligonucleotide microarrays [24,42]. To evaluate the significance of the mapping results, we carried out linkage analysis of 100 datasets with permutations of the segregant identities.

### Genetic analysis of *tlm* mutants.

Allelism of the *KanMx* locus and altered telomere length is evaluated by analysis of telomere length of tetrads of a cross between *MATa tlm :: KanMx* strain with wild-type *MATα* strain. Alternatively, we used a method for genetic selection of *MATa KanMx* progeny [18] and telomere length-analysis of random spores using either individual spores or pool of spores.

The analysis of *TLM* genes and genes in telomerase pathway *(TLC1* of *EST2)* and telomere-capping pathways (e.g., *YKU70*) was carried out by comparing telomere lengths of single *tlm, est2,* and *yku70* and double *tlm est2* or *tlm yku70* strains. For this analysis we replaced *EST2* and *YKU70* genes with a *NAT* gene that confers resistance to nursotrecine in a *MATα* strain that also contains a cassette for genetic selection of mating type [18] (Y3655 *MATα* *met15 ura3 lys2 leu2 can1::* MFA1-HIS3 MFalpha-*LEU2*). After mating with *MATa tlm :: KanMx* and sporulation, double *tlm est2* (or *tlm yku70*) haploid mutants were obtained through genetic selection of *MATa KanMx NAT* spores. Strains were allowed to grow for 20–25 divisions prior to telomere length-analysis. To control for the number of divisions following sporulation, single *tlm* and *yku70* and *est2* mutants were obtained using the described scheme after mating *tlm* strains or *est2* or *yku70* strains with the wild-type strains and selecting *MATa* haploid progeny with the *KanMx* (for *tlm* mutants) or *NAT* (for *yku70* and *est2* mutants).

For the effect of *TLM* genes on replicative senescence we carried out successive streak-outs of tetrads derived from *tlc1* and *tlm* heterozygous diploid strain *(MATa*/*α tlm :: KanMx*/*TLM tlc1::HIS3/TLC1).*

## Supporting Information

Figure S1 *TLM* Genes Affect Senescence Rate Independently of *RAD52*-Mediated Homologous Recombination Pathway Spores derived from *TLC1/tlc1 TML/tlm RAD52/rad52* heterozygous diploid strains were allowed to germinate, and haploid single *rad52*, double *rad52 tlc1*, *rad52* *tlm*, and *triple rad52 tlc1 tlm* mutants were streaked-out successively on rich medium. The strains with triple mutations (*rad52 tlc1 tlm*) grew poorly relative to strains containing *rad52 tlc1* double mutations.
(1.3 MB TIF)

Table S1 Candidate Telomere Length-Control Genes in BY × RM11 Cross (17 KB XLS)

## Abbreviations

bp: base pair
BY: laboratory strain
dNTP: deoxyribonucleotide
EST: ever shorter telomere gene
kb: kilobase
NMD: nonsense-mediating RNA decay
RM11: vineyard strain
TLM: telomere length-maintenance gene
VPS: vascuolar protein sorting
