# Correction: Telomere Length as a Quantitative Trait: Genome-Wide Survey and Genetic Mapping of Telomere Length-Control Genes in Yeast

**DOI:** 10.1371/journal.pgen.0020104

**Published:** 2006-06-30

**Authors:** Tonibelle Gatbonton, Maria Imbesi, Melisa Nelson, Joshua M Akey, Douglas M Ruderfer, Leonid Kruglyak, Julian A Simon, Antonio Bedalov

In *PLoS Genetics,* vol 2, issue 3: DOI:  10.1371/journal.pgen.0020035

Table 1 was incomplete. The full table is printed below. 

**Table 1:**
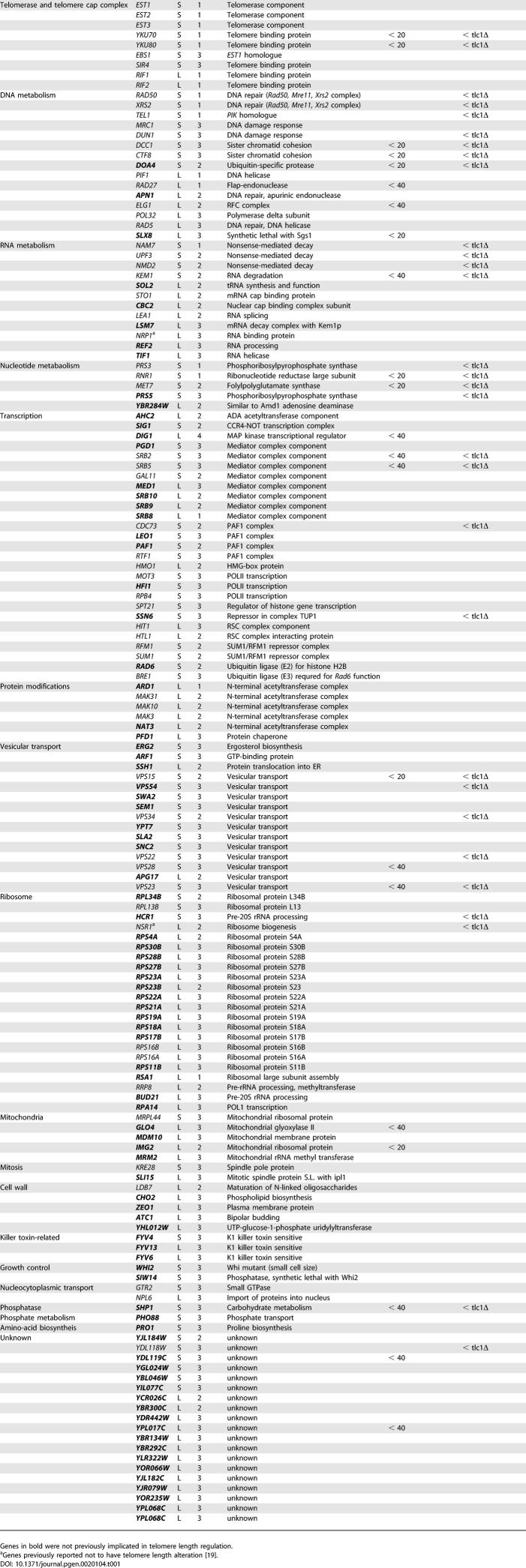
Genes Whose Deletion Affects Telomere Length and Their Interaction with Telomerase Pathway

